# Distal Radial Fractures in the Superelderly: Does Malunion Affect Functional Outcome?

**DOI:** 10.1155/2014/189803

**Published:** 2014-03-04

**Authors:** N. D. Clement, A. D. Duckworth, C. M. Court-Brown, M. M. McQueen

**Affiliations:** Department of Trauma and Orthopaedics, Royal Infirmary of Edinburgh, Little France, Edinburgh, EH16 4SA, UK

## Abstract

*Purpose*. The management of unstable distal radial fractures in the superelderly (≥80 years old) remains controversial. The aim of this study was to compare the functional outcome of super-elderly patients with and without malunion after a distal radial fracture. *Methods*. We identified 51 superelderly patients living independently with displaced fractures from a prospective database of 4024 patients with distal radial fractures. Activities of daily living, presence of wrist pain, whether the wrist had returned to its normal level function, grip strength and ROM were recorded. The dorsal angulation was measured radiographically. *Results*. There were 17 (33.3%) patients defined to have a malunion. The outcomes of the independent patients with and without malunion were compared at a mean follow-up of 15 months. No difference was observed in activities of daily living (*P* = 0.28), wrist pain (*P* = 0.14), whether the wrist had returned to its normal level function (*P* = 0.25), grip strength (*P* = 0.31), or ROM (*P* = 0.41). An increasing degree of dorsal angulation correlated with diminished ROM (*P* = 0.038), but did not correlate with activities of daily living (*P* = 0.10). *Conclusions*. Malunion of the distal radius does not influence the functional outcome of independent superelderly patients.

## 1. Introduction

The most prevalent fracture that trauma surgeons manage are those involving the distal radius [1], accounting for 16% of all fractures [2]. Nonoperative management is generally employed for stable nondisplaced fractures of the distal radius with the expectation of a good functional outcome [3–5]. In contrast, the management of displaced fractures of the distal radius remains controversial. Although some authors suggest that functional outcome correlates with the anatomical reduction of the fracture [4, 6–8] others suggest that this may not be the case [9–11]. This disparity may be due to the heterogeneity of the reported cohorts, which vary in size, have a lack of standardised reporting, and often combine both intra- and extra-articular fractures within the reported series [12]. In addition, multiple studies have reported cohorts with a wide age range; in one series, the age difference between the youngest and oldest patients was 80 years [3, 13–15]. However, age has been demonstrated to influence outcome [12] and therefore may have skewed the results of these studies.

It is predicted that there will be an increase in the elderly population over the next decade which is due to the 1950's Baby Boomers, and currently the fastest growing age group in the Western World is the “oldest old” (>85 years) [16]. It is anticipated that there will be an 81% increase in the Scottish population who are aged 75 years or more by 2031 [17]. The term “super-elderly” has been used in orthopaedics to describe those patients greater than 80 years of age [18, 19]. These superelderly patients account for approximately 20% of all distal radial fractures [20], which will likely increase in the future due to their growing population and will form a greater proportion of the orthopaedic workload.

The effect of a malunion upon the outcome of a distal radial fracture has been demonstrated to diminish with the increasing age [12]. Most studies reporting the outcome of distal radial fractures in the elderly, being defined as greater than 60 or 65 years of age, include low demand patients only [10, 21, 22]. The question remains as to whether a malunion results in an inferior outcome in superelderly patients due to their lower functional demands. Furthermore, the reduction of distal radial fractures has been shown to be of minimal benefit in frail elderly patients [10, 21, 22], and same could be asked of surgical intervention.

The primary aim of this study was to compare the functional outcome, both subjective and objective, of superelderly patients with and without malunion after a distal radial fracture. The secondary aim was to assess whether the final radiographic assessment of the distal radius correlated with range of motion and or function.

## 2. Materials and Methods

### 2.1. Demographic Data

A prospective database of 4024 distal radial fractures was compiled over a 67-month period at the study centre, which recorded the following: demographic, radiographic, management, and outcome of all patients. The mean age for all patients was 59 (14 to 100) years. Fifty-one patients who aged 80 years or older sustaining a displaced distal radial fracture with outcome data at one year and lived independently were retrospectively identified from this database and were defined as the study population. There were 50 females and one male with a mean age of 83.1 (80 to 93) years. Forty-eight patients (94%) were right-hand dominant. All fractures were unilateral.

### 2.2. Database Construction

Fracture management followed a standard protocol. The emergency room staff undertook the initial assessment and treatment. Fractures deemed to be in an acceptable position were managed with a dorsal plaster slab. If the fracture position was thought to be unacceptable, the emergency room staff, prior to application of a dorsal plaster slab, performed closed reduction using intravenous regional anaesthesia.

The patients were evaluated clinically and radiographically at approximately one and six weeks after the injury as per the protocol of the study unit, which included radiographs of the normal, uninjured wrist performed at one week.

At approximately one week following the injury, the patients were reviewed by the senior author in a dedicated research clinic. The clinical, demographic, and radiographic data were recorded and entered into a database either by the senior author or a research nurse. The premorbid normal level of function of the patients was categorised as independent if they were able to go shopping without assistance or as dependent if assistance was needed [23]. The patients with a fracture that had maintained a good position had the dorsal slab completed to a below-the-elbow forearm cast with the wrist in slight flexion and ulnar deviation. Patients with a fracture that had been displaced were admitted to the orthopaedic trauma unit for further intervention, unless the patient had low functional demands and operative intervention was deemed inappropriate.

The patients were subsequently evaluated at approximately six weeks and one year. Radiographs were repeated for the assessment of displacement. If surgical intervention had occurred, which was recorded, all radiographic measurements subsequent to surgery were used.

### 2.3. Radiographic Measurement Techniques

**Figure 1:**
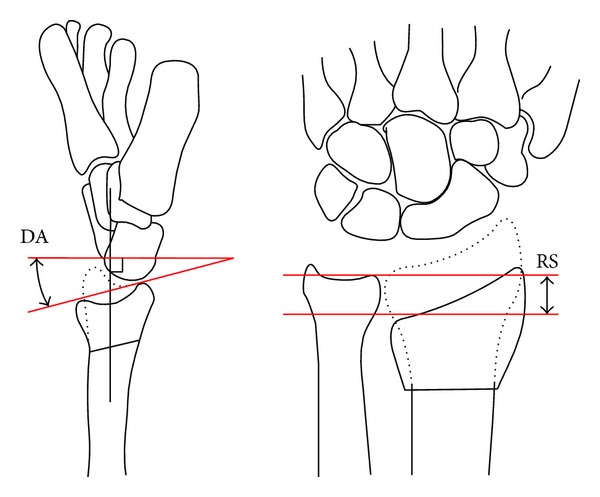
The measurement of dorsal angle (DA) and radial shortening (RS). These measurements were expressed as negative for volar angulation and positive for DA, and negative for RS.

All radiographs (presentation, time of reduction, one week, six weeks, and if preformed at one year) were measured manually with the use of a protractor and a ruler to provide values for the dorsal angle [24], and radial shortening [25]. These measurements are illustrated in Figure 1. The dorsal angle and radial shortening were expressed as the difference between the injured side and the normal uninjured side. If the normal values were unavailable or the patient had a prior fracture of the uninjured side (*n* = 2), the mean values for the normal side were used [26]. The fractures were classified using both the Frykman [27] and AO/OTA classifications [28]. The type of metaphyseal comminution was recorded, according to the location, as absent or as involving the dorsal metaphysis, volar metaphysis, or both the dorsal and volar metaphysis. Thus, comminution was a purely qualitative measurement. The senior author alone was responsible for fracture classification and the assessment of comminution. Malunion was defined as a dorsal angle of >10 degrees and or >3 mm of radial shortening [23].

### 2.4. Functional Assessment

Functional assessment was carried out by a single dedicated research physiotherapist at approximately one year after the index fracture. Objective measures assessed were range of movement (ROM) and grip strength and subjective measures assessed included the presence of pain at the wrist, if the wrist had regained its normal functional status for them, and whether they could perform a number of activities of daily living (see below).

ROM measured at the wrist and distal radioulnar joints were performed using a standard full circle goniometer [29, 30]. Intraobserver bias was minimised by careful technique and recordings were made in triplicate, and the mean of these measurements was recorded. The observer measured flexion, extension, pronation, supination, and radial and ulnar deviation for both the injured and uninjured sides. Grip strength was measured using a JAMAR Deluxe Hand Dynamometer, Model 0030J4 (Therapeutic Equipment Corporation, Clifton, New Jersey) [31–33]. In accordance with the guidelines for the use of this device, issued by the American Society for Surgery of the Hand, the mean of three successive readings was taken for each hand [34]. Each patient was examined at a similar time of the day at each assessment in order to minimise the effects of diurnal variation. The grip strength of the nondominant hand was increased by 10% for comparative analysis with the dominant side [31].

The presence or absence of pain was recorded for the injured wrist and whether they required analgesia because of their injury. Patients were also asked whether they felt their wrist had returned to the preinjury functional state. In addition, they were asked whether they could carry out a number of daily tasks: carry a plate, hold a glass, hold a pan, turn a key, bolt a door, and write and whether they could use scissors, a knife, a needle, and a hammer. Each of these ten tasks were assigned a score, one if they could not perform the task and two if they could; these scores were combined to give a total score for each patient, which is a validated assessment tool [34].

### 2.5. Statistical Analysis

SPSS version 16.0 software was used for statistical analysis (SPSS Inc., Chicago, IL, USA). Fisher's exact test was used to compare the dichotomous variables (activities of daily living, presence of wrist pain, and return to normal use) and an unpaired *t*-test was used to compare differences of liner variables (grip strength and ROM) between patients with and without malunion. Pearson's correlation coefficient was used to assess the relationship between dorsal angulation and radial shortening and ROM at the wrist. A *P* value of ≤0.05 determined statistical significance.

## 3. Results

Twenty-seven patients (52.9%) sustained a fracture of the right wrist and 24 patients (47.1%) sustained a fracture of the left wrist. The predominant mechanism was a fall from standing height (*n* = 48, 94.1%), and three patients (5.9%) fell down stairs. Forty-three patients (84.3%) were independent, with eight needing help to carry out their shopping. Tables 1 and 2 illustrate the distribution according to the OTA and Frykman classifications, respectively. Forty-two patients (82.4%) had dorsal comminution. The normal dorsal angle and ulna variance, of the uninjured side, were −8.3 degrees (SD 9.9 degrees) and +1.2 mm (SD 1.7 mm), respectively. The mean dorsal angulation was 16.1 degrees (0 to 44 degrees, SD 14.9) and radial shortening was 2.2 mm (−3 to 10 degrees, SD 2.6) for the injured side.

Thirty-five patients (68.6%) underwent manipulation within the emergency room setting, prior to application of a dorsal plaster slab. The pre- and postmanipulation radiographic measurements are shown in Table 3. However, 16 of these 35 (45.7%) lost their satisfactory position and underwent surgery. The final radiographic measurements for the 19 who did not undergo surgery are included in Table 3. Two (10.5%) of the 19 patients who underwent manipulation only, without a later surgical intervention, went on to malunion.

Eighteen (35.2%) patients underwent surgery of which 7 had open reduction internal fixation, 10 had an external fixator, and one patient had manipulation with insertion of Kirschner wires. The pre- and postoperative and final radiographic measurements are shown in Table 3. Four (22.2%) patients suffered minor pin tract infections, which resolved after oral antibiotics. Eight of the 18 (44.4%) had a malunion.

**Figure 2:**
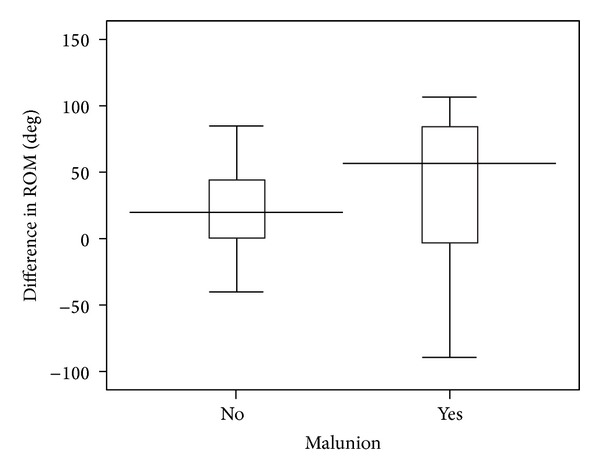
A box plot illustrating the loss in ROM by the interquartile range for patients with and without malunion. The horizontal black line represents the median value.

Seventeen (33.3%) patients had a malunion. The outcomes of the independent patients with and without malunion are compared in Table 4 at a mean follow-up of 15 (6 to 20) months. No statistically significant difference was observed in activities of daily living, wrist pain, whether the wrist had returned to its normal level of function, grip strength, or ROM.  Figure 2 illustrates no significant difference in the total loss in ROM for those patients with and without malunion (*P* = 0.41). Only one (12.5%) of the eight dependent patients suffered a malunion (odds ratio (OR) 0.24, *P* = 0.24). If the dependent group was also included in the outcome analysis, the only statistically significant difference was observed for the ability to lift a pan of water (OR 4.9, *P* = 0.03).

**Figure 3:**
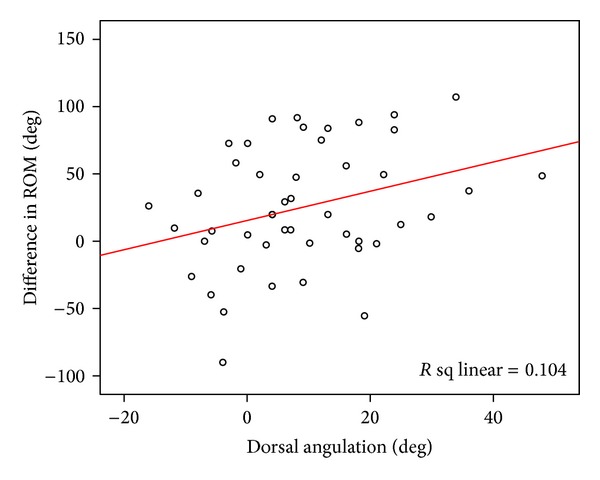
A scatter graph with a line of best fit showing the correlation between dorsal angle and global ROM for the wrist at final follow-up.

The final dorsal angle correlated significantly (*r* = 0.3, *P* = 0.038) with the ROM at the wrist (Figure 3), with diminished ROM being associated with increasing dorsal angulation. This correlation was not observed with radial shortening in isolation (*r* = 0.1, *P* = 0.46). In addition, there was no correlation between activities of daily living and dorsal angulation (*r* = 0.25, *P* = 0.10) or diminished ROM (*r* = 0.01; *P* = 0.95).

## 4. Discussion

This study has demonstrated that a malunion of the distal radius does not influence the functional outcome of independent superelderly patients. More than two-thirds of these patients were deemed to require manipulation of their displaced distal radial fracture, of which half went on to have surgery due to loss of reduction. A third of all patients underwent surgical intervention, which was associated with complications. Despite manipulation and surgical intervention, more than a quarter of patients still went on to malunion. The degree of malunion was illustrated to correlate with a reduced ROM, but neither the degree of malunion nor the associated diminished ROM influenced the functional outcome of the superelderly patients.

Colles [35] some 200 years ago on describing his fracture stated that “one consolidation only remains, that the limb at some remote period again enjoy perfect freedom in all its motions, and be completely exempt from pain: the deformity, however, will remain undiminished through life.” This statement may not have been fully supported by our results, as we observed a diminished ROM and some residual pain and dysfunction after a distal radial fracture in our superelderly cohort. Although the freedom of motion that Colles described may not relate to the absolute degree of movement, but to the freedom of motion would allow functional use of the limb. If this was his intention, our superelderly group supports his statement as it would seem that malunion, the persistent deformity he describes, does not hinder activities of daily living in this low functional demand group.

The correlation between malunion and functional outcome in elderly patients has been described; with no association being demonstrated for low demand patients with malunion union after a distal radial fracture and their functional outcome [10, 21, 22]. Beumer and McQueen [22] questioned whether reduction of displaced distal radial fractures should be attempted in very elderly, frail, dependent, or demented patients after finding that the majority (53/60) lost reduction and went on to malunion. Young and Rayan [21] and Chang et al. [10] illustrated that malunion did not correlate with poor functional outcome. However, these studies only included elderly patients, being 60 years or more, with low physical demands. More recently, Grewal and MacDermid [12] included all patients, with no exclusions according to physical demands and found no difference in the outcome of extra-articular fractures of the distal radius after malunion in patients greater than 65 years old. They did however demonstrate an increased risk of a poor functional outcome, defined as Disabilities of Arm Shoulder and Hand (DASH) score of greater than 20, with a malunion regardless of age, but this risk diminished with advancing age. However, the DASH score is not validated for patients at the extremes of age [36], and to state that a DASH score of 20 points or more is a poor outcome for very elderly patients is difficult to support as this score may be normal for them. In fact, one study found the mean DASH score to be 22 points for a group of patients with a mean age of 78 years after sustaining a distal radial fracture [37]. This supports our results for the superelderly population, with malunion having no influence upon functional outcome.

If the predicted increase of the superelderly population is correct, then they will form an increasing percentage of the orthopaedic trauma workload. This will have associated cost implications for both the management of their fracture and the need for increased social support while recovering from their fracture. The management of distal radial fractures, being the most prevalent fracture of the superelderly [20], will form the greatest proportion of the emergency room and orthopaedic trauma workload. If the results of our study are acknowledged, superelderly patients with a displaced distal radial fracture could be managed conservatively, without the need to reduce their fracture or to surgically intervene. These patients would not have to suffer the further discomfort of manipulation of their fracture or surgical measures with associated risks and still achieve a satisfactory functional outcome. This would also have cost saving implications, avoiding the need for primary reduction within the emergency room and the costs of surgery and reducing the number of clinic appointments and radiographs performed. This management protocol would also benefit the superelderly population, who would therefore endure less medical consultations and interventions but achieve an adequate functional outcome.

If a conservative protocol was followed for all distal radial fractures in the superelderly group, a potential risk would be the development of a symptomatic malunion in some patients. A distal radial osteotomy is indicated in fit patients with symptomatic malunion interfering with function irrespective of age [38–41]. Patients generally achieve a good functional outcome, but the rate of metalwork removal is high, from 25% to 54%, when plates are used to stabilise the osteotomy [38–41]. However, more recently, the use of a nonbridging external fixator has been described to stabilise the osteotomy, offering a minimally invasive technique and good functional results without the subsequent need to remove the metalwork [42]. This technique could be offered to those superelderly patients who develop a symptomatic malunion, if conservative methods fail to provide a satisfactory functional outcome.

There are several limitations to this study. The major limitation is the retrospective nature of this study and the small cohort analysed. However, the prospective data capture was of high quality, with only a single data point being absent (ROM of opposite wrist) for a single patient. In addition, this is the only case series reporting the outcome for superelderly (≥80 years) patients in the current literature. We also included both extra- and intra-articular fractures which may have skewed our results. However, on post hoc analysis, no statistical difference was observed between extra-articular (AO/OTA type A) and intra-articular (AO/OTA type B and C) fractures for rate of malunion, ROM, or functional outcome. A prospective randomised controlled trial comparing conservative versus interventional (manipulation or surgery) management would need to be performed to confirm our results before our proposed treatment protocol could be confidently recommended.

## 5. Conclusion

The limited functional demand of the superelderly population needs to be acknowledged before they are offered reduction of their distal radial fracture. Malunion of the distal radius, despite our best efforts to restore normal anatomical alignment, often occurs, but there would seem to be no functional deficit if it does occur for independent superelderly patients. This questions whether we should intervene after a displaced distal radial fracture in this population and suggests that we could manage these patients conservatively with the option of radial osteotomy in the small numbers of patients whose malunion may become symptomatic. This would have major repercussions in how superelderly patients with displaced distal radial fractures are managed, potentially avoiding the risks associated with fracture manipulation and surgical intervention but achieving the same functional outcome.

## Figures and Tables

**Table 1:** OTA class distribution for the 51 patients.

Classification	Frequency (%)
A2	3 (5.9)
A3	25 (49.0)
B3	4 (7.8)
C2	16 (31.4)
C3	3 (5.9)

Total	51 (100.0)

**Table 2:** Frykman class distribution for the 51 patients.

Classification	Frequency (%)
1	9 (17.6)
2	3 (5.9)
3	3 (5.9)
4	1 (2.0)
5	9 (17.6)
6	7 (13.7)
7	4 (7.8)
8	12 (23.5)
Unknown	3 (5.9)

Total	51 (100.0)

**Table 3:** Radiological evaluation of patients undergoing manipulation or surgical intervention.

Intervention	Time point	Dorsal angulation (SD)	*P* value^†^	Ulna variance (SD)	*P* value
Manipulation *n* = 35	Original	23.0 degrees (11.4)	—	−2.5 mm (2.4)	—
	After manipulation	0.2 degrees (9.7)	<0.0001	0.9 mm (1.7)	<0.0001
	Final*	6.8 degrees (14.5)	<0.0001	3.4 mm (2.8)	<0.0001

Surgery *n* = 18	Original	21.2 degrees (13.1)	—	−2.3 mm (2.1)	—
	After surgery	6.6 degrees (6.0)	<0.0001	2.8 mm (2.6)	<0.0001
	Final	12.9 degrees (11.7)	<0.0001	1.8 mm (2.4)	<0.0001

*19 patients only, as 16 of the 35 went on to have surgery, ^†^paired *t*-test.

**Table 4:** Comparison of subjective and objective outcome variables for independent patients with and without malunion.

Outcome variable	Malunion		Odds ratio or 95% CI	*P* value
	Yes *n* = 16	No *n* = 27		
Activities of daily living				
Able to:				
Plate	80.0%	96.0%	2.3	0.14^†^
Glass	100%	100%	—	—
Pan	66.7%	91.7%	4.6	0.10^†^
Key	100%	100%	—	—
Bolt	100%	100%	—	—
Write	93.8%	100%	2.8	0.37^†^
Scissors	100%	100%	—	—
Knife	100%	96.2%	1.6	0.62^†^
Needle	86.7%	91.3%	1.2	1.0^†^
Hammer	93.8%	96.2%	1.4	1.0^†^
Total ADL score	**19.0**	**19.3**	−**0.9 to 0.28**	0.28^††^
Wrist pain	**18.8%**	**3.7%**	**6.0**	0.14^†^
Normal use	**43.8%**	**59.2%**	**1.5**	0.25^†^
Grip strength*	**−2.0**	**−4.1**	−**2.0 to 6.1**	0.31^††^

ROM* (degrees)				
Pronation	−5.8	−0.6	−15.3 to 14.6	0.15^††^
Supination	−5.1	−2.5	−11.6 to 6.4	0.56^††^
Flexion	−20.7	−9.5	−21.4 to 0.34	0.85^††^
Extension	0.0	−3.1	−6.7 to 13.1	0.52^††^
Radial deviation	−2.5	0.0	−9.3 to 4.3	0.47^††^
Ulna deviation	−3.3	−7.9	−4.0 to 13.3	0.93^††^
Global	36.8	22.5	−15.0 to 43.5	0.41^††^

*Difference compared with opposite (normal) wrist.

^†^Fisher's exact test.

^††^unpaired *t*-test.
